# Redox Regulation in Cancer Stem Cells

**DOI:** 10.1155/2015/750798

**Published:** 2015-07-26

**Authors:** Shijie Ding, Chunbao Li, Ninghui Cheng, Xiaojiang Cui, Xinglian Xu, Guanghong Zhou

**Affiliations:** ^1^Key Lab of Meat Processing and Quality Control, College of Food Science and Technology, Nanjing Agricultural University, Nanjing 210095, China; ^2^USDA/ARS Children Nutrition Research Center, Department of Pediatrics, Baylor College of Medicine, Houston, TX 77030, USA; ^3^Departments of Surgery and Obstetrics and Gynecology, Women's Cancer Program, Samuel Oschin Comprehensive Cancer Institute, Cedars Sinai Medical Center, Los Angeles, CA 90048, USA

## Abstract

Reactive oxygen species (ROS) and ROS-dependent (redox regulation) signaling pathways and transcriptional activities are thought to be critical in stem cell self-renewal and differentiation during growth and organogenesis. Aberrant ROS burst and dysregulation of those ROS-dependent cellular processes are strongly associated with human diseases including many cancers. ROS levels are elevated in cancer cells partially due to their higher metabolism rate. In the past 15 years, the concept of cancer stem cells (CSCs) has been gaining ground as the subpopulation of cancer cells with stem cell-like properties and characteristics have been identified in various cancers. CSCs possess low levels of ROS and are responsible for cancer recurrence after chemotherapy or radiotherapy. Unfortunately, how CSCs control ROS production and scavenging and how ROS-dependent signaling pathways contribute to CSCs function remain poorly understood. This review focuses on the role of redox balance, especially in ROS-dependent cellular processes in cancer stem cells (CSCs). We updated recent advances in our understanding of ROS generation and elimination in CSCs and their effects on CSC self-renewal and differentiation through modulating signaling pathways and transcriptional activities. The review concludes that targeting CSCs by manipulating ROS metabolism/dependent pathways may be an effective approach for improving cancer treatment.

## 1. Introduction

Reactive oxygen species (ROS), including superoxide (O_2_^−^), hydrogen peroxide (H_2_O_2_), and hydroxyl radical (OH^•^), are highly chemically reactive species derived from molecular oxygen [1, 2]. Under physiological conditions, ROS are generated as byproducts from the mitochondrial electron transport chain [2]. ROS can also be produced by various oxidases, such as NADPH oxidases and peroxidases, in different cellular compartments or organelles, such as cell membranes, peroxisomes, and endoplasmic reticulum [3]. Furthermore, chemotherapy, radioactivity, and even smoking can increase ROS levels in the cell [4–6]. The low-to-moderate ROS level in the cell will generally promote cell proliferation and growth and increase cell survival [7]. On the contrary, when in excess, ROS can cause cellular toxicity and trigger apoptosis [8, 9]. The antioxidant systems in the cell can scavenge ROS and prevent irreversible cellular oxidative damage [10]. Therefore, it is important for cells to balance ROS generation and antioxidant systems, and redox regulation of cellular process is essential for growth and development.

ROS levels are elevated in many cancer cells partially due to their higher metabolism rate [11, 12]. Aberrant ROS levels can elicit cancer cell apoptosis and necrosis [13]. Cancer cells have high antioxidant capacity to counteract and scavenge ROS. Because high antioxidant capacity enhances cell survival and impairs cellular responses to anticancer therapy [14], induction of ROS-mediated damage in cancer cells by proper pharmacological agents that either promote ROS generation beyond the cellular antioxidative capacity or disable the cellular antioxidant system has been considered as a “radical” therapeutic strategy to preferentially kill cancer cells [14].

In recent years, the concept of cancer stem cells (CSCs) has been gaining ground as the subpopulation of cancer cells with stem cell-like properties and characteristics have been found and reported in various cancers, including leukemia [15], breast cancer [16], and pancreatic cancer [17]. CSCs are thought to have the ability to self-renew and differentiate [1] and be responsible for cancer recurrence after chemotherapy or radiotherapy as those cells can survive treatment and then quickly generate new tumors [18, 19]. These abilities of CSCs lead to a view that cancer therapy strategies should target not only the normal cancer cells, but also the CSCs.

Considering the importance of redox balance in cancer cells, conventional therapies (chemotherapy or radiotherapy) targeting redox balance can kill most of the cancer cells [14, 20, 21]. However, the unique redox balance in CSCs and its underlying mechanisms to protect CSCs from ROS-mediated cell killing have not been fully understood [22–24]. In this review, we will update the effects of ROS/redox regulation on the properties and functions of CSCs. With special attention given to the cross talk between CSC-related pathways and redox regulation, we hope to generate substantial interest in further investigating the role of redox regulation in CSCs and the utility of targeting ROS-dependent/redox regulation of pathways.

## 2. ROS Production and Scavenging in CSCs

In cancer cells, ROS are mainly generated through high-rate metabolism at mitochondria, endoplasmic reticulum, and cell membranes [3]. The metabolic phenotypes observed in tumor cells are different from the normal tissue, which are attributed to the Warburg effect [25–28]. The glycolysis replaces at least part of the oxidative phosphorylation for generation of ATP in cancer cells [28]. This metabolic switch is essential for the cancer cells to adapt to hypoxic conditions with less mitochondrial defects and ROS production [20].

The CSCs, similar to normal stem cells, are quiescent, slow-cycling cells with the lower level of intracellular ROS [29, 30], which accounts for their self-renewal capacity and resistance to chemotherapy drugs and ionizing radiation [29]. For example, in human gastrointestinal cancer cells, the stem-like population (CD^44^ high) has lower ROS levels [31]. CSCs in some human and murine breast tumors also have lower ROS levels [29]. This lower ROS level in CSCs could be attributed to less ROS production and/or enhanced ROS scavenging systems. The slow division of CSCs may generate less ROS than regular cancer cells. Indeed, Dey-Guha et al. reported that rapidly proliferating breast cancer cells could produce slowly proliferating “G0-like” progeny by asymmetric division [32]. The “G0-like” cancer cells behave like the stem cells in “quiescent” state and may be able to maintain a stable “out of cycle” state for a long period of time* in vivo* [32]. Intracellular ROS contents and AKT expression are lower in these cells [32].

Many signaling pathways and transcriptional activity contribute to scavenging ROS in normal stem cells and CSCs as well (see details in the following sections). Among them, forkhead homeobox type O (FOXO) is essential for maintaining low ROS levels in haematopoietic stem cells (HSCs), which are critical for the stemness of HSCs [33]. Furthermore, ataxia telangiectasia mutation (ATM) can upregulate the antioxidant enzymes and downregulate the differentiation and proliferation genes, as a result to help maintain the low ROS levels and the stemness [24]. In pancreatic cancer stem cells, activation of JNK pathway is important for their maintenance of stemness and resistance to drugs, 5-fluorouracil and gemcitabine, through suppressing ROS generation induced by those chemotherapeutic agents [34].

Recently, Diehn et al. investigated how CSCs maintained the lower ROS levels [29]. It was found that the ROS were reduced due to upregulation of free radical scavenging systems, such as glutathione (GSH) [29]. Furthermore, Nagano et al. showed that expression of one of the CD44^+^ variant isoforms (CD44v) in CSCs contributed to upregulation of GSH biosynthesis. The CD44v protein may promote cysteine uptake by interacting with and stabilizing the xCT, which is the subunit of the cysteine-glutamate transporter xc(-). This process leads to increased GSH synthesis [35]. Recent studies indicate that the epigenetic regulation may also play an important role in the regulation of ROS in CSCs. The downregulation of fructose-1,6-biphosphatase (FBP1) by epigenetic mechanisms increased the rate of glycolysis but decreased the ROS level in basal-like breast cancer, resulting in the activation of *β*-catenin signaling to maintain CSCs [36]. MicroRNA may also play an important role in the regulation of ROS production/scavenging in CSCs [37, 38].

## 3. ROS-Dependent Signaling Pathways in CSCs

### 3.1. PTEN/PI3K/AKT/mTOR Pathway

The PI3K pathway is commonly activated in human cancers. Numerous studies have demonstrated that the PI3K pathway plays a prominent role in cancer cell growth and survival [39]. The activated PI3K/AKT/mTOR signaling pathway can also increase cell metabolism and glycolysis, which in turn affects the intracellular ROS level and tumorigenesis [40, 41]. Phosphatase and tensin homolog deleted on chromosome 10 (PTEN), a major negative regulator of PI3K, is a tumor suppressor [42]. PTEN encodes a lipid phosphatase that converts phosphatidylinositol 3,4,5-trisphosphate (PIP3) to phosphatidylinositol 4,5-bisphosphate (PIP2). PIP3 is necessary for the downstream activation of AKT. PTEN mutations can lead to PIP3 accumulation and as a result overactivate the AKT pathway [43, 44]. The mutation or deletion of PTEN is well known to be involved in the development of many cancers [45, 46].

In CSCs, PI3K/AKT signaling pathway is upregulated. During neovascularization, CSCs can function as initiators of tumor neovascularization [47]. They can produce proangiogenic factors and transdifferentiate into vascular mural cells and form nonendothelium-lined vasculogenic mimicry [47]. Activation of the PI3K/AKT signaling pathway can induce vascular endothelial growth factor (VEGF) production in CD133^+^ glioma stem-like cells [48]. VEGF, in turn, induces angiogenesis and vasculogenesis by driving the transdifferentiation of CSCs [48]. Consistent with this, another study showed that activation of the PI3K/AKT pathway was required for breast cancer stem-like cell maintenance [49]. On the other hand, inhibition of PI3K/AKT/mTOR activity by NVP-BEZ235 (the dual PI3K/mTOR inhibitor) led to a decrease in the CD133^+^/CD44^+^ stem-like populations [50]. PTEN also plays a critical role in CSCs. Its expression is lower in recurrent hepatocellular carcinoma [51]. Furthermore, the upregulation of the miR-216a/217 cluster, which targets PTEN [51, 52], downregulates PTEN and elicits epithelial-mesenchymal transition (EMT) and cancer stem-like properties in hepatocellular carcinoma [51]. PTEN deletion contributes to the depletion of normal HSCs but increases the generation of leukemia-initiating cells. This brings a rare distinction in PTEN regulation in the maintenance of normal stem cells compared with leukemia-initiating cells [53]. PTEN knockdown by shRNA leads to an increase in sphere formation for enriching prostate cancer stem-like cell as well as increases in clonogenic and tumorigenic potential [50].

In CSCs, regulation of the PTEN/PI3K/AKT/mTOR signaling pathway can be ROS-dependent/redox regulation. Higher H_2_O_2_ treatment (100 *μ*M) can induce the phosphorylation of AKT and activate its activity in glioma-initiating cells [54]. In CSCs, ROS-dependent oxidized cellular environment is important in modulating the catalytic activity of PTEN. H_2_O_2_ may abrogate PTEN activity through inducing the formation of a disulfide bond between the active sites Cys^124^ and Cys^71^, while Trx may reduce oxidized PTEN to reactivate it [55].

The PTEN/PI3K/AKT/mTOR signaling pathway in CSCs could control cellular ROS levels through regulating nuclear-localized FOXOs [29]. The FOXOs regulate the production of MnSOD and catalase to scavenge ROS [56]. Dey-Guha et al. reported that, in ER^+^/HER^2−^ human breast cancer MCF7 cell line, the ROS^low^ cancer cells had higher levels of nuclear-localized FOXO1 [32]. Furthermore, the repression mTOR will inhibit hypoxia-inducible factor-1*α* (HIF-1*α*) translation in hypoxic conditions [57]. The transcriptional targets of HIF-1*α* contain VEGF and FOXOs which are related to the stemness and ROS removal [58].

### 3.2. ATM Pathway

ATM is critical for maintaining genome stability. It can regulate DNA damage repair, particularly for double-strand breaks [24]. ATM upregulates the glucose-6-phosphate dehydrogenase to promote NADPH production and thus reduces the ROS level [59]. In CSCs, ATM signaling pathway is highly active. In CD44^+^/CD24^−^ stem-like cells compared to other cell populations from breast cancer cell lines and breast tumors, the expression of ATM was significantly increased [60]. The ATM inhibitor reversed the radiation resistance of CD44^+^/CD24^−^ cells, which suggests the importance of ATM signaling in CSCs [60].

### 3.3. Notch Pathway

The Notch pathway is critical for a series of processes, including cell fate specification, differentiation, proliferation, survival, and apoptotic programs [61]. It is essential for the maintenance of stem cells, such as neural stem cells and HSCs [62–64]. However, this pathway is also very important in CSCs. Recent evidence showed that HIF-1*α*-induced activation of the Notch pathway was essential for hypoxia-mediated maintenance of glioblastoma stem cells [65]. McAuliffe et al. demonstrated that the Notch signaling pathway, Notch3 in particular, was critical for ovarian CSC survival and platinum resistance [66]. Notch3 overexpression in ovarian tumor cells resulted in expansion of CSCs and platinum chemoresistance. On the contrary, *γ*-secretase inhibitor, a Notch pathway inhibitor, or Notch3 siRNA knockdown, increased tumor sensitivity to platinum [66]. Besides Notch3, Notch1 and Notch2 also protected glioma stem-like cells against radiation. Knockdown of Notch1 or Notch2 sensitized glioma stem-like cells to radiation and impaired xenograft tumor formation [67]. These results confirm the significance of Notch signaling in CSCs.

The Notch pathway is critical for controlling the ROS level in CSCs. One possible target is the PI3K/AKT pathway. Prosurvival factor AKT is upregulated by Notch in glioma stem cells [65]. The PI3K/AKT pathway will later upregulate the ROS scavenging enzymes. On the other hand, ROS can also stimulate the Notch signaling pathway in order to maintain the CSCs. The nitric oxide released by endothelial cells can activate Notch signaling and promote the stemness of the PDGF-induced glioma cells [68]. Charles et al. showed that nitric oxide pathway enhanced the side population phenotype in cultured human glioma cells through activation of Notch signaling [68].

### 3.4. Wnt Pathways

Wnt signaling is important in embryo development and also controls homeostatic self-renewal in adult tissues [69]. Radioresistant breast cancer cells showed CSC-like properties and elevation of *β*-catenin. NS398, a cyclooxygenase 2 inhibitor, enhanced the radiosensitivity of these cells, which may be partially via downregulating the expression of *β*-catenin [70].

High levels of ROS can inhibit *β*-catenin activation [36, 71]. Nucleoredoxin, a Trx family protein, was found to interact with disheveled, which was important in Wnt signaling [72]. In line with this finding, H_2_O_2_ inhibited the association between disheveled and nucleoredoxin, blocking the Wnt-*β*-catenin pathway [72]. Recent studies indicated that, in basal-like breast cancer stem cells, overexpression of FBP1 enhanced oxidative phosphorylation and ROS production and decreased *β*-catenin signaling by promoting its dissociation from TCF4 [73]. However, whether Wnt signaling is directly involved in this metabolic regulation remains for further investigation.

### 3.5. STAT Pathway

STAT3 is highly expressed in solid tumor and is involved in the formation of nitric oxide to promote cell survival [74]. In head and neck squamous cell carcinoma, CD44^+^ALDH1^+^ cells are tumorigenic and radioresistant [75]. Interestingly, cucurbitacin 1, a STAT3 inhibitor, effectively inhibited the tumorigenicity, sphere formation, resistance to radiation, and BCL-2 expression in these cells [75]. STAT signaling is also activated in non-small cell lung cancer, in which CD133^+^ stem-like cells showed high p-STAT3 levels compared to CD133^−^ cells. Inhibition of STAT3 by cucurbitacin 1 decreased the p-STAT3 level and the CD133^+^ population, while increasing apoptosis [76].

In contrast, in breast cancer cells, the STAT3 is redox-sensitive and H_2_O_2_ decreases STAT3 binding to the consensus serum-inducible elements with inhibition of cell proliferation and reduced survival [77]. The STAT3 pathway can be positively regulated by mTOR signaling in human breast cancer stem-like cells [49]. The PTEN is found as a negative regulator of both STAT3 and mTOR [49]. The ROS effects on CSCs by STAT3 signaling may be mediated through the PTEN/PI3K/ATK/mTOR signaling.

Other signaling pathways may also regulate ROS in CSCs. The p-ERK was found to be higher in CD133^+^ human hepatocellular carcinoma compared to CD133^−^ cells. Further studies showed that the lower ROS levels were related to ERK activation and were important for the radioresistance of CD133^+^ cells [78]. The p38 MAPK signaling can be activated by ROS. In glioma-initiating cells, H_2_O_2_ induced ROS can increase p38. The upregulated p38 will induce Bmi1 protein degradation and FOXO3 activation, leading to the differentiation [54].

## 4. ROS-Dependent Transcription Factors in CSCs

### 4.1. HIF

The HIF family transcriptional factors are upregulated in hypoxia [79]. Hypoxia is a well-recognized microenvironmental condition in stem cells and CSCs [1, 58, 65, 80]. HIFs have an oxygen-sensitive HIF*α* subunit and a constitutively expressed HIF*β* subunit. Under normoxic conditions, HIF*α* could be targeted for proteasomal degradation with the Von Hippel-Lindau (VHL) tumor suppressor gene product. In hypoxia condition, the interaction between HIF*α* and VHL is abrogated. Then the stabilized HIF*α* could dimerize with HIF*β* and then induce transcription of its target genes [81, 82]. HIF*α* has 3 isoforms and recent studies have demonstrated that HIF-1*α* and HIF-2*α* play a critical role in CSCs. Li et al. found that HIF-2*α* was highly expressed in glioma stem cells (GSCs) and its regulated genes were preferentially expressed in comparison to nonstem tumor cells and normal neural progenitors [82]. As compared to growth at 20% oxygen level, tumor stem-like cells (CD133^+^ cells) from human glioblastoma grown at 7% oxygen level show an increase in the expression levels of the neural stem cell markers CD133 and nestin as well as the stem cell markers Oct4 and Sox2 [83]. HIF-1*α* is not affected in CD133^+^ tumor stem-like cells grown at 7% oxygen level but HIF-2*α* is expressed at higher levels as compared with that at 20% oxygen level [83]. However, the hypoxia (1% oxygen) promotes the self-renewal capacity of CD133^+^ CSCs by upregulation of HIF-1*α* in glioma stem cells [84].

Some studies indicate that ROS can regulate HIF*α* expression. HIF-1*α* has been found to mediate EGF-induced prostate cancer cell EMT phenotype [85] and STAT3 downstream of ROS is implicated in EGF-induced HIF-1*α* transcription and protein expression [85]. Another study indicated that increased level of intracellular ROS in well-oxygenated conditions, but not hypoxia, was a causative factor of the transient upregulation of HIF-1 activity during the metastatic colonization of cancers in the lungs [86]. One possible reason is that the Fe^2+^ is essential for the prolyl hydroxylation of HIF-1*α* by prolyl hydroxylase domain proteins (PHDs) and the PHDs-VHL-proteasome is important for HIF-1*α* stability. However, the Fe^2+^ could be oxidized by the ROS [86]. Further studies found that the HIF-1*α*-mediated metabolic reprogramming (mitochondrial oxidative phosphorylation to anaerobic glycolysis and lactic acid fermentation) reduced ROS levels and increased the survival of metastatic cancers [86].

### 4.2. NF-*κ*B

The transcription factor NF-*κ*B plays a critical role in cell survival, proliferation, immunity, and inflammation [87]. NF-*κ*B has been widely studied in breast cancer and acute myelogenous leukemia (AML) and other cancers for chemotherapy resistance [88]. Once activated, it will induce the expression of a variety of cell survival factors to prevent apoptosis. NF-*κ*B regulation is important in CSCs. Inhibition of NF-*κ*B in mammary epithelial cells may reduce tumor stem cell marker expression and CSC populations [89]. Parthenolide, a sesquiterpene lactone, can block NF-*κ*B, leading to the death of AML progenitor and stem cell population and a decrease of engraftment* in vivo *[90]. It is suggested that parthenolide may render these cells sensitive to oxidative stress [90]. NF-*κ*B activation triggered by RAC1 and ROS production is important in colorectal cancers initiation [91].

There is an extensive cross talk between ROS and NF-*κ*B signaling. Morgan and Liu showed that ROS may regulate NF-*κ*B activation to express antioxidant genes coding manganese superoxide dismutase (MnSOD, or SOD2), copper-zinc superoxide dismutase (Cu, Zn-SOD, or SOD1), catalase, and Trx [92]. These enzymes can directly or indirectly scavenge ROS and protect cells from ROS-induced cytotoxicity. However, in immune cells, activated NF-*κ*B may regulate Nox, resulting in elevated production of ROS [93]. In the cytoplasm, oxidizing conditions may cause I*κ*B*α* degradation and NF-*κ*B activation, while, in the nucleus, a reducing environment is necessary for DNA binding and transcriptional activity of NF-*κ*B dimmers [94].

Considering the low ROS levels, the upregulation of NF-*κ*B in CSCs may contribute to redox balance. NF-*κ*B suppresses ROS- and/or JNK-mediated killing induced by oncogene products or anticancer agents [95]. In acute myelogenous leukemic stem cells (LSCs), quenching ROS by the GSH precursor,* N*-acetylcysteine, will weaken the niclosamide-induced apoptosis. The niclosamide (an antineoplastic) may inhibit the TNF*α*-induced NF-*κ*B activation and increase the intracellular ROS levels [96].

### 4.3. p53

The p53 plays an important role in protecting normal cells from cancer development. Almost all human cancers lost the activity of p53 [97]. In CSCs of nasopharyngeal carcinoma, treatment by resveratrol suppressed the CSC properties including resistance to therapy and self-renewal, tumor initiation, and metastatic potential [98]. Mechanistically, resveratrol impeded CSC functions through the activation of p53 and knocking down p53 could reverse this effect. In addition, resveratrol exploited p53 to suppress stemness and EMT [98]. In an ErbB2 transgenic model of breast cancer, the p53 in mammary stem cells was found to regulate the cell division polarity and the knockout of p53 induced the symmetric divisions of CSCs and tumorigenesis [99]. Furthermore, treatment of leukemia CSCs with selenium would increase ROS levels and induce the apoptosis via the activation of the ATM-p53. This treatment would not affect hematopoietic stem cells [100]. The inhibition of NF-*κ*B, activation of p53 and increased ROS levels by parthenolide can induce the apoptosis of LSCs in AML [90].

The p53 can regulate genes that generate or scavenge ROS and can exert pro- and antioxidant effects depending on its levels [101]. Sablina et al. found that the prooxidant function of p53 was due to release of mitochondrial ROS during stress-induced apoptosis. But the antioxidant function of p53 was related to the expression of antioxidant gene products, which were responsive to lower levels of p53 in no stressed or physiologically stressed cells [101]. On the other hand, ROS can also regulate p53 activity via oxidation of p53 cysteine residues to inactivate p53 [102]. The cross talk between p53 and ROS signaling is of great importance in cell cycle and apoptosis regulation [102].

### 4.4. Nrf2

The nuclear factor erythroid 2-related factor (Nrf2) is a key regulator of defense against endogenous and exogenous stresses by governing expression of many antioxidant and detoxification genes [103]. In normal cells, Nrf2 binds to the inhibitor protein Keap1 [104]. But in many cancer cells, loss of Keap1 function activates Nrf2 and promotes cancer growth [105]. Nrf2 is a key factor to inhibit the differentiation of glioma stem-like cells, and the knockout of Nrf2 may promote the differentiation process [106].

Nrf2-regulated antioxidant genes include GSH synthesis and GSH reductase and peroxidase families [107]. In a secretome analysis of colon CSCs, there is a significant overlap between the set of proteins in the secretome and those that are regulated by transcription factor Nrf2, which suggests that, in CSCs, activation of the Nrf2-antioxidant pathway protects them from oxidative stress [108]. In mammospheres, which are thought to enrich breast cancer cells with stem/progenitor features, the Nrf2-mediated cellular protective response is induced under the taxol treatment. Inhibition of the Nrf2 pathway enhanced intracellular ROS levels and rendered mammospheres more sensitive to taxol [109].

## 5. Antioxidant Proteins in CSCs

### 5.1. Trx

The Trx system contains the redox-active protein Trx, thioredoxin reductase (TrxR), and NADPH. This system is important for cellular functions especially for protection against oxidative stress [110]. Three Trxs, including Trx1, Trx2, and spTrx (specifically expressed in human spermatozoa), have been identified in mammalian cells. All of them contain a conserved -Cys-Gly-Pro-Cys- active site. This site is essential for disulfide oxidoreductase [110, 111]. The Trx1 and Trx2 are similar in structure and catalytic mechanism. TrxRs catalyze Trxs through the NADPH-dependent reduction of the disulfide. The C-terminus of reduced TrxRs possesses the high reactivity of selenide, which can help the balance of redox [112]. In the cell, the endogenous inhibitor of Trx1 is the thioredoxin-interacting protein (TXNIP), which is dramatically downregulated in various human cancers [113].

In cancer cells, high proliferation results in high ROS production [20, 114, 115]. To maintain redox homeostasis, cancer cells also produce high levels of antioxidant proteins. In non-small cell lung cancer, Trx and TrxR are highly expressed [116]. Ceccarelli et al. derived cell clones with different levels of Trx from the same lung carcinoma cell lines. It was found that high level of Trx correlated with invasive and metastatic potentials of the cells [117]. A significant correlation exists between tumor resistance to docetaxel and Trx expression in breast cancer patients [118]. A recent study showed that a histone methyltransferase inhibitor killed CD34^+^CD38^−^ leukemia stem cells by reactivating TXNIP and inhibiting Trx activity [119]. These results suggest Trx may be critical for CSC function.

### 5.2. Grx

Glutaredoxin (Grx) system is another important redox system in cells. It was firstly discovered in Trx-mutant* Escherichia coli* that show a fully active NADPH-dependent ribonucleotide reductase system [120]. Grxs are small heat-stable oxidoreductase [121]. Grxs catalyze thiol-disulfide exchange reactions with GSH, glutathione reductase (GR), and NADPH. The Grx is reduced via GSH within the Grx system, while the GSH disulfide is reduced by GR and NADPH [122]. Besides the maintenance of cellular redox environment, Grxs are involved in the maintenance of cytosolic and mitochondrial iron homeostasis [122, 123].

In breast cancer cells, Grx1 overexpression can cause adriamycin-resistance [124]. Recently, two human testis-specific isoforms of Grx2, Grx2b and Grx2c, are abnormally expressed in various cancer cell lines [125]. In human cancer cells, Grx overexpressed cells showed the resistance to glucose deprivation-induced cytotoxicity. Glucose deprivation induces the ROS stress and activates the ASK1-SEK1-JNK1 signaling causing cytotoxicity [126]. Whether Grxs play an essential role in CSCs remains to be determined.

### 5.3. Prdx

Peroxiredoxins (Prdxs) are a group of peroxidases that consist of one or two redox-active cysteine residues and reduce peroxides with conserved cysteine residues [127], six isoforms of which are present in mammalian tissues (Prdx1–Prdx6) that play a role in cellular protection against oxidative stress [127].

Expression of Prdxs is upregulated under oxidative stress. Prdx1 has been proposed as a potential breast cancer marker [128]. It was reported that the increased Prdx6 activity promotes the growth of lung cancer cells and enhances the metastatic potential of lung cancer cells [129]. The Prdx3 is upregulated in many endocrine-regulated tumors, such as prostatic intraepithelial neoplasia [130]. In the antiandrogen-resistant cell lines, increased Prdx3 enhanced resistance to H_2_O_2_ [130]. The knockout of Prdx3 can trigger the proapoptotic signals with antiandrogen and H_2_O_2_ treatment [130].

## 6. ROS Regulation in Therapeutical Implication

CSCs has been found to exist in different cancers, including AML, breast, brain, head and neck, pancreas, lung, prostate, colon, and sarcoma cancers. In cancer treatment, the chemotherapy and radiation therapies are widely used but the patients invariably relapse. The CSCs are always dormant, which can help its resistance to conventional chemotherapies that brings cytotoxicity to dividing cells [131]. CSCs keep lower ROS level with overexpression of antioxidant enzymes, which can help them survive from chemotherapy and radiation induced ROS [132, 133].

Considering the importance of ROS in CSCs, ROS regulation is also significant in therapy resistance as chemotherapy and radiation therapy affect ROS levels. Phillips et al. found that CD24^−/low^/CD44^+^ breast cancer stem/initiating cells were resistant to radiotherapy and possessed low ROS levels [5]. Similarly, prostate CSCs contained more low-to-intermediate ROS-producing cells after ionizing radiation [134]. After chemotherapy, CD13^+^ liver CSCs decreased the ROS level by expressing a scavenger enzyme CD13/aminopeptidase N [133]. Chemotherapeutic drugs can also generate ROS and DNA double-strand breaks in cancer treatment. In the chemoresistant case, the ROS/SUMO (small ubiquitin-like modifier) axis is not activated. The sensitivity of LSCs can be achieved by inhibiting the ROS/SUMO pathway [135].

Interfering with intracellular redox balance for selectively killing the cancer cells is becoming a hot topic in therapeutical treatment. Lagadinou et al. found that these ROS^low^ LSCs overexpressed BCL-2. Inhibition of BCL-2 decreased levels of GSH, which could increase the oxidative state and selectively eradicate quiescent LSCs [136]. In treatment with glioblastoma multiforme, the inhibitors of GSH synthesis can potentiate TMZ- (DNA alkylating agent temozolomide-) induced bystander effect [137]. Brusatol, an inhibitor of the Nrf2 pathway, downregulates the protein level of Nrf2 and its target genes. As a result, it sensitizes mammospheres to taxol [109]. Deregulation of miRNAs related to ROS is also a new therapeutic approach in cancer treatment [38]. The ROS induces miR-200 family expression and further downregulating ZEB1, which is likely to play a key role in ROS-induced apoptosis and senescence [138]. The induction of ROS and the inhibition of the Nrf2 and HIF-1*α* pathways can also decrease the colony-forming ability of LSC-like cells and apoptosis [139]. A new drug, fenretinide, has been developed to directly target AML-stem cells. The drug can induce AML-stem cells death by rapid generation of ROS, upregulation of the stress responses and apoptosis related genes, and downregulation of the genes in NF-*κ*B and Wnt signaling [140].

Recent studies showed that the shikonin (a TrxR1 inhibitor) could induce apoptosis mediated by ROS in human promyelocytic leukemia HL-60 cells. The chemical broke the ROS balance by targeting the selenocysteine residue in TrxR1 and blocked its physiological function [112]. The 3-deazaneplanocin A can reactivate TXNIP, which in turn inhibits the Trx activity and increases the level of ROS. As a result, it leads to the apoptosis in AML cell lines, primary cells, and CD34^+^CD38^−^ LSCs [119].

## 7. Conclusions

While there is limited information on ROS regulation in CSCs, there is fast emerging evidence that ROS may play an essential role in the self-renewal and differentiation ability of CSCs. ROS-dependent signaling pathways and transcriptional activities control redox balance and ROS regulation in CSCs. Targeting CSCs via ROS regulation and antioxidant proteins holds great potential in improving cancer therapy.

## Abbreviations

CSCs: Cancer stem cells
ROS: Reactive oxygen species
O_2_^−^: Superoxide
H_2_O_2_: Hydrogen peroxide
OH^•^: Hydroxyl radical
NADPH: Reduced nicotinamide adenine dinucleotide phosphate
HSCs: Haematopoietic stem cells
EMT: Epithelial-mesenchymal transition
PIP3: Phosphatidylinositol 3,4,5-trisphosphate
PIP2: Phosphatidylinositol 4,5-bisphosphate
HIF-1: Hypoxia-inducible factor-1
PTEN: Phosphatase and tensin homolog deleted on chromosome 10
LSCs: Leukemic stem cells
SODs: Superoxide dismutases
Nrf2: Nuclear factor erythroid 2-related factor
GSH: Glutathione
ATM: Ataxia telangiectasia mutation
PI3K: Phosphoinositide 3-kinase
EMT: Epithelial-mesenchymal transition
JNK: C-Jun N-terminal kinase
AML: Acute myelogenous leukemia
Trx: Thioredoxin
TrxR: Thioredoxin reductase
Grx: Glutaredoxin
Prdx: Peroxiredoxins
VEGF: Vascular endothelial growth factor
TXNIP: Thioredoxin-interacting protein
COX: Cyclooxygenase
FOXO: Forkhead homeobox type O
PHDs: Prolyl hydroxylase domain proteins.
